# In vivo quantitative MRI: *T*_*1*_ and *T*_*2*_ measurements of the human brain at 0.064 T

**DOI:** 10.1007/s10334-023-01095-x

**Published:** 2023-05-20

**Authors:** Kalina V. Jordanova, Michele N. Martin, Stephen E. Ogier, Megan E. Poorman, Kathryn E. Keenan

**Affiliations:** 1 Physical Measurement Laboratory, National Institute of Standards and Technology, NIST, Boulder, CO USA; 2 Department of Physics, University of Colorado Boulder, Boulder, CO USA; 3 Hyperfine, Inc., Guilford, CT USA

**Keywords:** Magnetic resonance imaging

## Abstract

**Objective:**

To measure healthy brain ${T}_{1}$ and ${T}_{2}$ relaxation times at 0.064 T.

**Materials and methods:**

${T}_{1}$ and ${T}_{2}$ relaxation times were measured in vivo for 10 healthy volunteers using a 0.064 T magnetic resonance imaging (MRI) system and for 10 test samples on both the MRI and a separate 0.064 T nuclear magnetic resonance (NMR) system. In vivo ${T}_{1}$ and ${T}_{2}$ values are reported for white matter (WM), gray matter (GM), and cerebrospinal fluid (CSF) for automatic segmentation regions and manual regions of interest (ROIs).

**Results:**

${T}_{1}$ sample measurements on the MRI system were within 10% of the NMR measurement for 9 samples, and one sample was within 11%. Eight ${T}_{2}$ sample MRI measurements were within 25% of the NMR measurement, and the two longest ${T}_{2}$ samples had more than 25% variation. Automatic segmentations generally resulted in larger ${T}_{1}$ and ${T}_{2}$ estimates than manual ROIs.

**Discussion:**

${T}_{1}$ and ${T}_{2}$ times for brain tissue were measured at 0.064 T. Test samples demonstrated accuracy in WM and GM ranges of values but underestimated long ${T}_{2}$ in the CSF range. This work contributes to measuring quantitative MRI properties of the human body at a range of field strengths.

**Supplementary Information:**

The online version contains supplementary material available at 10.1007/s10334-023-01095-x.

## Introduction

The field of quantitative magnetic resonance imaging (qMRI) is concerned with extracting characteristic biomarkers from a magnetic resonance (MR) image that have physical units attached to them [1, 2]. Methods for qMRI have been developed for measuring length and volume, relaxation properties (${T}_{1}$, ${T}_{2}$, ${T}_{2}$*), fluid flow, diffusion, phase, fat fraction, proton density, etc. To achieve clinical utility of these methods, accurate knowledge of quantitative values for healthy and diseased tissue is critical [3, 4].

There has been renewed interest in MRI at field strengths ≤ 0.55 T for point-of-care diagnostics due to lower cost, greater portability, quieter operation, improved implant safety, reduced susceptibility artifacts, increased accessibility, and reduced limitations on the specific absorption rate while imaging compared to conventional MR systems (1.5 T and 3 T) [5]. Several scanners that operate at field strengths ≤ 0.55 T are in development [6–15]. Many companies have products available or under development at lower field strengths, including at 0.064 T (Swoop, Hyperfine, Guilford CT, USA), 0.066 T (Promaxo Inc., Oakland CA, USA), 0.345 T (MRIdian Linac, ViewRay, Mountain View CA, USA), 0.4 T (Magnifico Open, Esaote, Genoa, Italy), 0.5 T (Synaptive Medical Inc., Toronto, Canada), 0.55 T (MAGNETOM Free.Max, Siemens Healthineers, Erlangan, Germany), and at undisclosed field strengths (neuro42, San Francisco CA, USA). A natural extension of lower field MRI is to adopt qMRI methods, for which accurate knowledge of quantitative tissue properties at the relevant field strength is crucial. In the past, many in vivo quantitative measurements at field strengths ≤ 0.55 T have suffered from hardware limitations, causing trade-offs between feasibility and accuracy [16, 17]. Although there has been increased investigation into healthy brain relaxation parameters at lower field strengths [18], additional measurements are necessary for each specific field strength and hardware, and for ensuring reproducibility of qMRI measurements.

We measure ${T}_{1}$ and ${T}_{2}$ relaxation parameters in the brain using a commercially-available 0.064 T MRI system. We design and test the in vivo measurement protocols by measuring test sample relaxation times on the 0.064 T MRI system and comparing them to 0.064 T NMR ${T}_{1}$ and ${T}_{2}$ measurements. ${T}_{1}$ and ${T}_{2}$ tissue values for white matter (WM), gray matter (GM), and cerebrospinal fluid (CSF) are reported.

## Materials and methods

### Measurement systems

Measurements were conducted on a 0.064 T Hyperfine Swoop MRI scanner (hardware 1.8, software rc8.3.1, Guilford CT, USA) using an 8-channel receive, 1-channel transmit head coil. The study was conducted in accordance with IRB guidelines, and all subjects provided informed consent. Additionally, the National Institute of Standards and Technology Research Protections Office reviewed and approved the research protocol, and the study was performed in accordance with the ethical standards as laid down in the 1964 Declaration of Helsinki and its later amendments.

Relaxation time measurements for a set of test samples were made on a Bruker variable field electromagnet nuclear magnetic resonance (NMR) system set to a field strength of 0.064 T, using a Redstone spectrometer and TNMR software (Tecmag, Houston, TX, USA). A custom RF copper solenoid coil was used, which was designed to allow perfluorocarbon coolant (TMC Industries, Inc., Waconia, MN, USA PN: FC-40) to flow around the sample for temperature control.

### Test sample synthesis protocols

Test samples were prepared by dissolving stock solutions of metal compounds into deionized water. Specifically, the compounds used included CuSO_4_·5H_2_0 (Millipore-Sigma, St. Louis, MO, USA; Part number (PN): 209198), GdCl_3_·6H_2_0 (Millipore-Sigma, St. Louis, MO, USA; PN: 203289), edetic acid (EDTA) (Millipore-Sigma, St. Louis, MO, USA; PN: 324503), and NiCl_2_·6H_2_0 (Millipore-Sigma, St. Louis, MO, USA; PN: N6136). GdCl_3_-EDTA was made by stirring a GdCl_3_ and EDTA (at twice the mmol/L value of GdCl_3_) solution on a 98 °C hotplate for 30 min. Dry agarose (Millipore-Sigma, St. Louis, MO, USA; PN: A6013) was weighed and added to the paramagnetic salt solution, followed by heat cycles: (1) 30 s interval microwave cycle until boiling; (2) 10 min hotplate cycle to ensure well-hydrated agarose. Deionized water was added to the mixture to make up for mass lost to evaporation. The mixture was poured into 50 ml or 30 ml sample tubes, pre-washed with isopropyl alcohol.

The selected test samples were: 0.1 mmol/L CuSO_4_, 2 mmol/L CuSO_4_, 0.75 mmol/L CuSO_4_ in 0.25% agarose mass concentration (%), 1 mmol/L CuSO_4_ in 1% agarose mass concentration (%), 0.025 mmol/L GdCl_3_-EDTA, 0.1 mmol/L GdCl_3_-EDTA in 1.5% agarose mass concentration (%), 4 mmol/L NiCl_2_ in 1.2% agarose mass concentration (%), 0.1% agarose mass concentration (%), 0.5% agarose mass concentration (%), and deionized water. These samples were chosen for their expected similarity in ${T}_{1}$ and ${T}_{2}$ to WM, GM, or CSF at 0.064 T.

### Quantitative imaging protocols

#### Optimization of ${T}_{1}$ mapping protocol via simulation

${T}_{1}$ maps were acquired using an inversion recovery (IR) sequence, chosen for its robustness in ${T}_{1}$ measurements compared to other ${T}_{1}$ mapping methods [19].

The ${T}_{1}$ measurement protocol was optimized to acquire accurate values for WM, GM, and CSF in a feasible scan time for in vivo use. The repetition time ($TR$) and inversion times ($TI$s) were chosen by the following optimization process: (1) simulating the expected IR signal in the presence of noise; (2) solving for ${T}_{1}$ using the noisy simulations and IR signal equation; and (3) choosing the protocol that minimized the error in estimated ${T}_{1}$.

Specifically, MR signals were simulated for ${T}_{1}$ values in the range of 0.1 s to 4 s using the IR signal equation [20]:

1 $${S}_{i}={S}_{0}\left|1-\left(1+d\right){e}^{-\frac{{TI}_{i}}{{T}_{1}}}+{e}^{-\frac{TR}{{T}_{1}}}\right|+{n}_{i}$$

with ${S}_{i}$ as the simulated signal for the $i$th $TI$, scale factor for imperfect inversion $d$ (set to 0.95), and nominal signal intensity for a voxel ${S}_{0}$. A total of 25 simulations of Eq. 1 with additive Rician noise (${n}_{i}$) [21] were used to estimate ${T}_{1}$, from which a mean ${T}_{1}$ estimate was calculated. The nominal signal magnitude ${S}_{0}$ was varied such that the signal-to-noise ratio (SNR) of the simulated signals ranged from 5 to 95. An SNR of 95 corresponds to the SNR observed in IR scans of the test samples. The ranges of ${TI}_{i}$ and $TR$ that were tested were selected such that the total scan time for the ${T}_{1}$ mapping protocol would be less than one hour.

${T}_{1}$ was estimated from the simulated signals ${S}_{i}$ for each [${TI}_{i}$, $TR$] combination, and the optimal protocol was chosen such that it minimized simulated ${T}_{1}$ error for a range of SNR values, especially for the target tissue ${T}_{1}$ values (as given in [18]). While there was not one protocol with optimal performance for all SNR values, the optimal protocol was selected for particularly good performance for SNR between 50 and 95, and reasonably good performance for SNR down to 25. Below an SNR of 25, all protocol simulations degraded substantially in their ${T}_{1}$ estimation capability.

#### ${\mathrm{T}}_{1}$ mapping protocol

${T}_{1}$ Maps were acquired using a research version of the Hyperfine ${T}_{1}$-weighted IR 3D fast spin echo (FSE) sequence.

The optimized protocol resulted in a $TR$ of 2.4 s and $TI$s of 0.05 s, 0.15 s, 0.35 s, 0.5 s, 0.95 s, 1.995 s. Images were acquired with a 1.6 mm^2^ in-plane resolution and 5 mm slice thickness, with a field of view of 22 cm × 18 cm × 18 cm. Each IR scan time was 9 min and 46 s, leading to a total IR session time of 58 min and 48 s, not including pre-scan calibration and localizer sequences. The total scan session time was typically around 61 min.

#### ${\mathrm{T}}_{2}$ mapping protocol

To acquire ${T}_{2}$ maps, a research version of a Hyperfine ${T}_{2}$-weighted 3D FSE sequence with 10 echo times ($TE$s) was used. The protocol had a $TR$ of 3 s and $TE$s of 0.037 s, 0.111 s, 0.185 s, 0.259 s, 0.333 s, 0.407 s, 0.480 s, 0.554 s, 0.628 s, 0.702 s, and was acquired on a spiral-out k-space trajectory with two dummy echoes. Images were acquired with a 1.5 mm^2^ in-plane resolution and 5 mm slice thickness, with a field of view of 22 cm × 18 cm × 18 cm. The ${T}_{2}$ mapping sequence scan time was 17 min and 8 s, not including pre-scan calibration and localizer sequences. A typical total scan session time was around 20 min.

### MRI measurements

#### In vivo measurements

In vivo ${T}_{1}$ and ${T}_{2}$ measurements were acquired using the protocols described above from 10 healthy volunteers (5 male, 5 female, ranging from 20 to 56 years old). Due to the extended scan time for each imaging protocol and to minimize strain on volunteers, quantitative measurements were acquired over two sessions for each volunteer. The average inter-session time was 9.3 days, with 8 volunteers having an inter-session time of less than or equal to 8 days, and two volunteers having longer inter-session times of 29 days and 42 days.

#### NMR measurements

${T}_{1}$ and ${T}_{2}$ measurements were acquired for the test samples using the in vivo protocols described above on the 0.064 T MRI system, using a custom Hyperfine-provided phantom that held the 50 ml sample tubes. Additionally, ${T}_{1}$ measurements were acquired on the 0.064 T MRI system using a reference protocol with 29 $TI$s that is prohibitively long for in vivo scanning ($TI$s of 0.1 s, 0.15 s, 0.2 s, 0.25 s, 0.3 s, 0.35 s, 0.4 s, 0.45 s, 0.5 s, 0.55 s, 0.6 s, 0.7 s, 0.8 s, 0.9 s, 1.0 s, 1.1 s, 1.2 s, 1.3 s, 1.4 s, 1.5 s, 1.6 s, 1.7 s, 1.8 s, 1.9 s, 2.0 s, 2.1 s, 2.2 s, 2.3 s, 2.4 s; $TR$ of 2.8 s; 1.6 mm^2^ in-plane resolution; 5 mm slice thickness; and total imaging time of approximately 5.5 h).

For the ${T}_{1}$ NMR measurements, an IR sequence was used with 20 exponentially increasing $TI$s in steps of [0.001*x to *x*], with *x* being sample-dependent and ranging from 2.5 s to 15 s, selected such that the largest inversion time was longer than 5 times the expected ${T}_{1}$. For the ${T}_{2}$ NMR measurement, a Carr-Purcell-Meiboom-Gill (CPMG) sequence was used with 20 linearly increasing $TE$s in steps of [0.05*x to *x*] with *x* being sample-dependent and ranging from 0.08 s to 3.76 s, selected such that the longest echo time was 2.5 times to 3 times the expected ${T}_{2}$. For both ${T}_{1}$ and ${T}_{2}$, a final delay time of 5 times the expected ${T}_{1}$ was used. The samples for the NMR measurements were kept at 21.3 °C, the approximate temperature of the laboratory housing the 0.064 T MRI system.

### Quantitative parameter analysis

#### Quantitative parameter map reconstruction

For all quantitative parameters, we assumed one quantitative value per voxel, and partial volume effects were not taken into account.

${T}_{1}$ was calculated for each voxel using least squares minimization (lmfit, Python) for the IR model mentioned earlier [20]:

2 $${S}_{i}={S}_{0}\left|1-\left(1+d\right){e}^{-\frac{{TI}_{i}}{{T}_{1}}}+{e}^{-\frac{TR}{{T}_{1}}}\right|$$

where ${T}_{1}$ is the target value for the fit. For long ${T}_{1}$ and in the presence of noise, the in vivo protocol is ill-conditioned and results in very large estimated error in the ${T}_{1}$ measurements, on the order of the measurement itself. Thus, in vivo ${T}_{1}$ measurements were excluded when the standard error of the fit exceeded the measurement itself. A total of 14 voxels out of 80,389 total voxels were excluded.

The ${T}_{2}$ map was calculated for the Hyperfine protocol using non-linear least squares optimization (SciPy optimize curve_fit, Python) for the model:

3 $${S}_{i}={S}_{0}{e}^{-\frac{TE}{{T}_{2}}}$$

where ${T}_{2}$ is the target value for the fit. A monoexponential decay was assumed and used to fit Eq. 3 directly to the image-space data.

#### Test sample region of interest selection

For ${T}_{1}$ and ${T}_{2}$ maps of the test samples acquired with the 0.064 T MRI system, regions of interest (ROIs) were selected using an automated protocol that searched for circles of the expected 50 ml tube size in each image slice. Once each tube was identified for each slice, the ROI of each tube was limited to a central circular region of half the tube’s image radius. ${T}_{1}$ was fit using data from all voxels located in each ROI simultaneously, which resulted in one ${T}_{1}$ per ROI and per slice. ${T}_{2}$ values came directly from the Hyperfine software, and the mean of the ${T}_{2}$ value for all voxels in each ROI was calculated to give one ${T}_{2}$ value per ROI per slice. Finally, the six central slices of the phantom were selected due to their clean ROI tube segmentations compared to slices near the ends of the tubes, which had ROI segmentations that sometimes included the ends of the tube. The final reported ${T}_{1}$ and ${T}_{2}$ values represent the mean and standard deviation of each tube’s ROI over these central six slices.

For 0.064 T NMR measurements, three repeated measurements were acquired for both ${T}_{1}$ and ${T}_{2}$, and the reported mean and standard deviation for each sample were calculated over the three replicate measurements. ${T}_{2}$ was calculated using Eq. 3, whereas because the NMR measurements used $TR$ > 5*${T}_{1}$, ${T}_{1}$ was calculated using a simplified form of Eq. 2, namely:

4 $${S}_{i}={S}_{0}\left|1-\left(1+d\right){e}^{-\frac{{TI}_{i}}{{T}_{1}}}\right|$$

#### In vivo image segmentation

For in vivo images, ${T}_{1}$ and ${T}_{2}$ were calculated on a per-voxel basis using Eqs. 2, 3 for a slice chosen to be approximately centered on the ventricles, such that the anterior horn is well visualized. The skull was stripped away from each in vivo map using a thresholding-open-close image transformation series of operations. For ${T}_{1}$, the raw IR images were used in 8 of 10 volunteers to aid in the skull stripping.

A segmentation algorithm inspired by O’Reilly & Webb [18] was implemented to distinguish WM, GM, and CSF. A mixture of Gaussians was used with Python Scikit-learn Gaussian Mixture (GMM) to model the acquired ${T}_{1}$ or ${T}_{2}$ measurements, and voxels were binned into one of the tissue compartments based on the ${T}_{1}$ or ${T}_{2}$ value. To attempt to account for potential partial volume mixture between CSF and GM, a “partial” volume bin was included in the segmentation algorithm for tissue segmentation. Additionally, as in previous work [18], ${T}_{2}$ was indistinguishable for WM and GM using the auto-segmentation mixture of Gaussians method, and it was thus segmented as one tissue.

Manual selection of ROIs for WM, GM, and CSF were created via visual inspection of the raw images, the relaxation parameter maps, and the automatic segmentations. Small regions of 2 voxels × 2 voxels were selected by locating visually dissimilar areas to attempt to identify WM from GM in the right frontal lobe. For CSF, a region was selected wherever the ventricles showed the highest relaxation values. Manual CSF ROIs were selected from the right side for 5 volunteers, from the left side for 5 volunteers. The automatic segmentation results were used as a reference such that manually selected ROIs only included voxels from one automatically segmented region.

### Statistical analysis

Relaxation time measurements for test samples using the NMR system versus the MRI system were compared by plotting the 95% confidence interval using the mean and standard deviation of each test sample, on each system. All MRI protocol measurements were normalized to the respective NMR measurement for each sample and relaxation parameter. Measurements that had overlapping 95% confidence intervals were said to be similar.

Bland–Altman plots were used to compare in vivo measurements from the manually selected ROIs to the measurements using the automatic segmentations. ${T}_{1}$ and ${T}_{2}$ were normalized to the average measurement value before calculating paired averages and differences for the Bland–Altman plots.

## Results

**Fig. 1:**
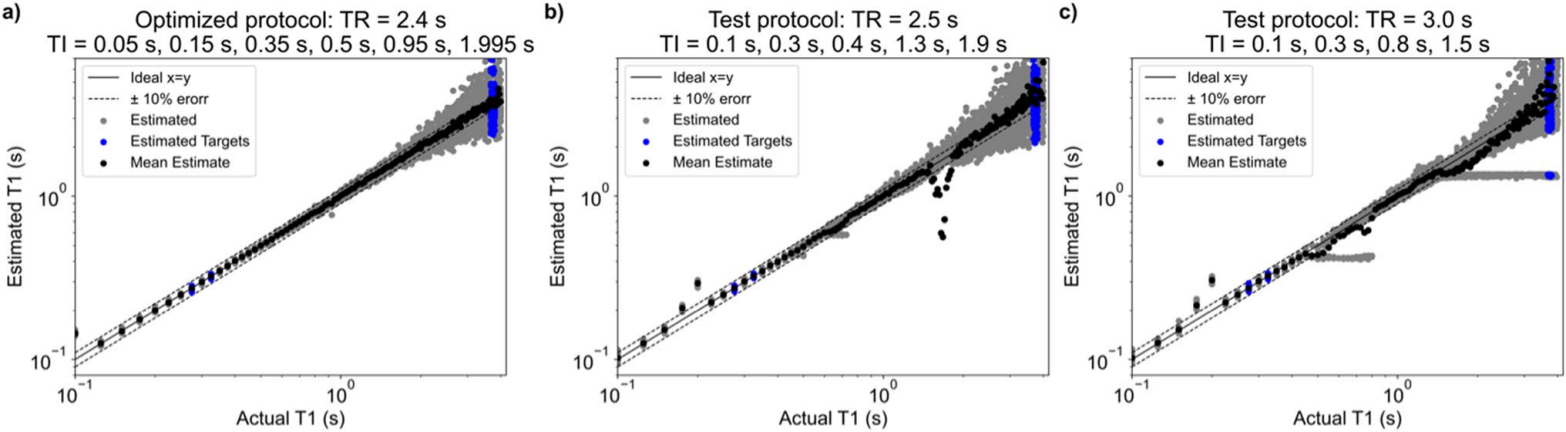
**a** Simulation results of estimated versus actual ${T}_{1}$ for the selected in vivo ${T}_{1}$ mapping protocol. Six $TI$ s were selected with a repetition time of 2.4 s, resulting in a total scan time of 60 min. Gray dots indicate each ${T}_{1}$ estimate of 25 simulations with added noise; black indicates the mean over the 25 noisy estimates; blue indicates estimated ${T}_{1}$ for the target expected ${T}_{1}$ values of WM, GM and CSF at 0.064 T. **b** Similar to plot (**a**), simulation results of an example sub-optimal ${T}_{1}$ mapping protocol that would greatly overestimate ${T}_{1}$ of around 0.2 s, and greatly underestimate ${T}_{1}$ around 2 s. **c** Similar test protocol to (**b**), but with a longer $TR$ and one fewer $TI$ s, resulting in worse ${T}_{1}$ estimation overall. SNR = 95 was used to simulate (**a**–**c**)

Simulation results of estimated versus actual ${T}_{1}$ were used to select an optimized ${T}_{1}$ protocol (Fig. 1a) and resulted in a mean error in ${T}_{1}$ estimation of 2.03%. For comparison, simulation results from two non-optimal test protocols had mean errors in ${T}_{1}$ estimation of 39.0% and 187% (Fig. 1b, c).

**Fig. 2:**
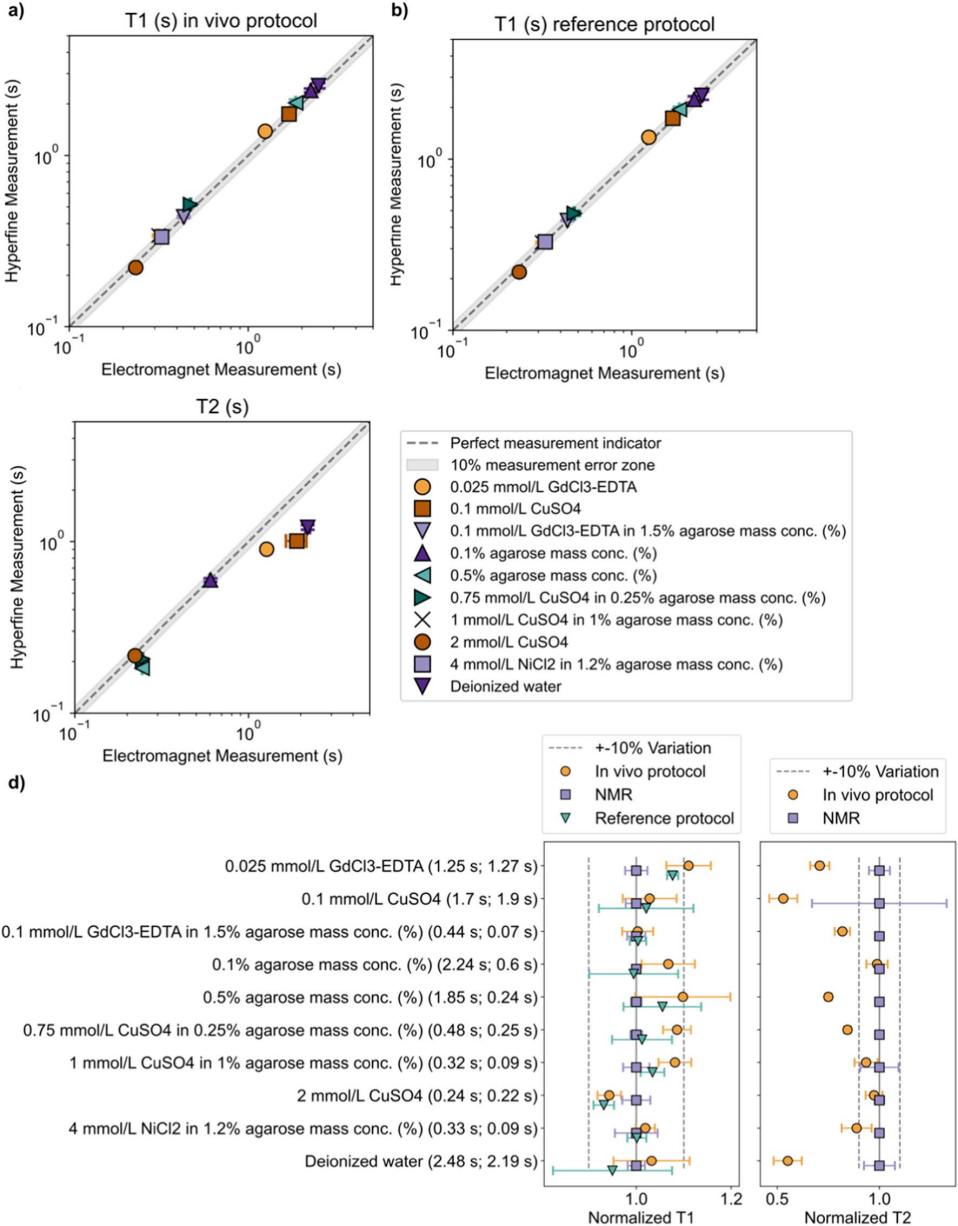
**a**–**c** Sample validation results for the Hyperfine scan protocols compared to measurements made on an NMR electromagnet set to 0.064 T. **a** ${T}_{1}$ test sample results using the in vivo MRI protocol (1 h scan time) compared to the NMR measurements. **b** ${T}_{1}$ test sample results using a reference MRI ${T}_{1}$ protocol (5.5 h scan time) compared to NMR measurements. **c** ${T}_{2}$ test sample in vivo MRI protocol results compared to NMR measurements. **d** Comparisons for test sample ${T}_{1}$ (left) and ${T}_{2}$ (right) measurements using the NMR measurements as a reference versus the MRI in vivo and reference protocols. Measured NMR (${T}_{1}$, ${T}_{2}$) values are shown next to each material label. Averages and 95% confidence intervals are plotted for each sample and measurement type

To better understand measurement uncertainty, test samples were used to compare ${T}_{1}$ and ${T}_{2}$ measurements from the MRI system to measurements made using the 0.064 T NMR (Fig. 2). The Supplementary Information Table S.1 lists the test sample mean and standard deviation for the measurements shown in Fig. 2. ${T}_{1}$ measurements using the optimized in vivo MRI protocol had mean difference from the NMR measurements of 5.82% (Fig. 2a) and was acquired in one hour. By comparison, a reference MRI protocol that used 15 $TI$s and was acquired in 5.5 h had mean difference from the NMR measurements of 3.28% (Fig. 2b). All ${T}_{1}$ measurements were within 10% of the NMR measurement, except for 0.025 mmol/L GdCl_3_-EDTA, which has an 11% difference between the NMR measurement and in vivo MRI protocol.

For the test samples, the ${T}_{2}$ measurements using the in vivo MRI protocol had a mean difference from the NMR measurements of 20.1% (Fig. 2c). Eight samples have ${T}_{2}$ MRI measurements that lie within 25% of the NMR measurement, with 3 samples having MRI measurements within 10% of the NMR measurements. Specifically, for ${T}_{2}$ ≤ 1.27 s, MRI and NMR measurements are within 25% of each other. For ${T}_{2}$ ≥ 1.27 s, the in vivo MRI ${T}_{2}$ mapping protocol greatly underestimates ${T}_{2}$ compared to the NMR measurements.

The test sample measurement uncertainty was further quantified using confidence intervals of the in vivo MRI protocol measurements compared to confidence intervals of the NMR system measurements (Fig. 2d). ${T}_{1}$ measurements using the NMR system and measurements using the reference MRI protocol had overlapping 95% confidence intervals for all samples except 0.025 mmol/L GdCl_3_-EDTA and 2 mmol/L CuSO_4_. Comparing the NMR system to the in vivo MRI protocol, 0.025 mmol/L GdCl_3_-EDTA, 2 mmol/L CuSO_4_, and 0.75 mmol/L CuSO_4_ in 0.25% agarose mass concentration (%) had non-overlapping 95% confidence intervals. Seven of 10 ${T}_{2}$ MRI measurements were not within the 95% confidence interval range of the NMR measurements.

The efficacy of the skull stripping algorithm is explored in Supplementary Information Fig. S.1. Example in vivo IR images from the optimized ${T}_{1}$ protocol are shown for one volunteer, and the full ${T}_{1}$ map for the same volunteer and the skull stripped version are shown for comparison, where the raw IR image for $TI$ of 0.05 s was used to aid in skull stripping for this volunteer. An example ${T}_{2}$ map and skull stripping result for a different volunteer are also shown. Based on qualitative observation, the skull stripping algorithm sufficiently removed the skull without affecting brain soft tissue.

**Fig. 3:**
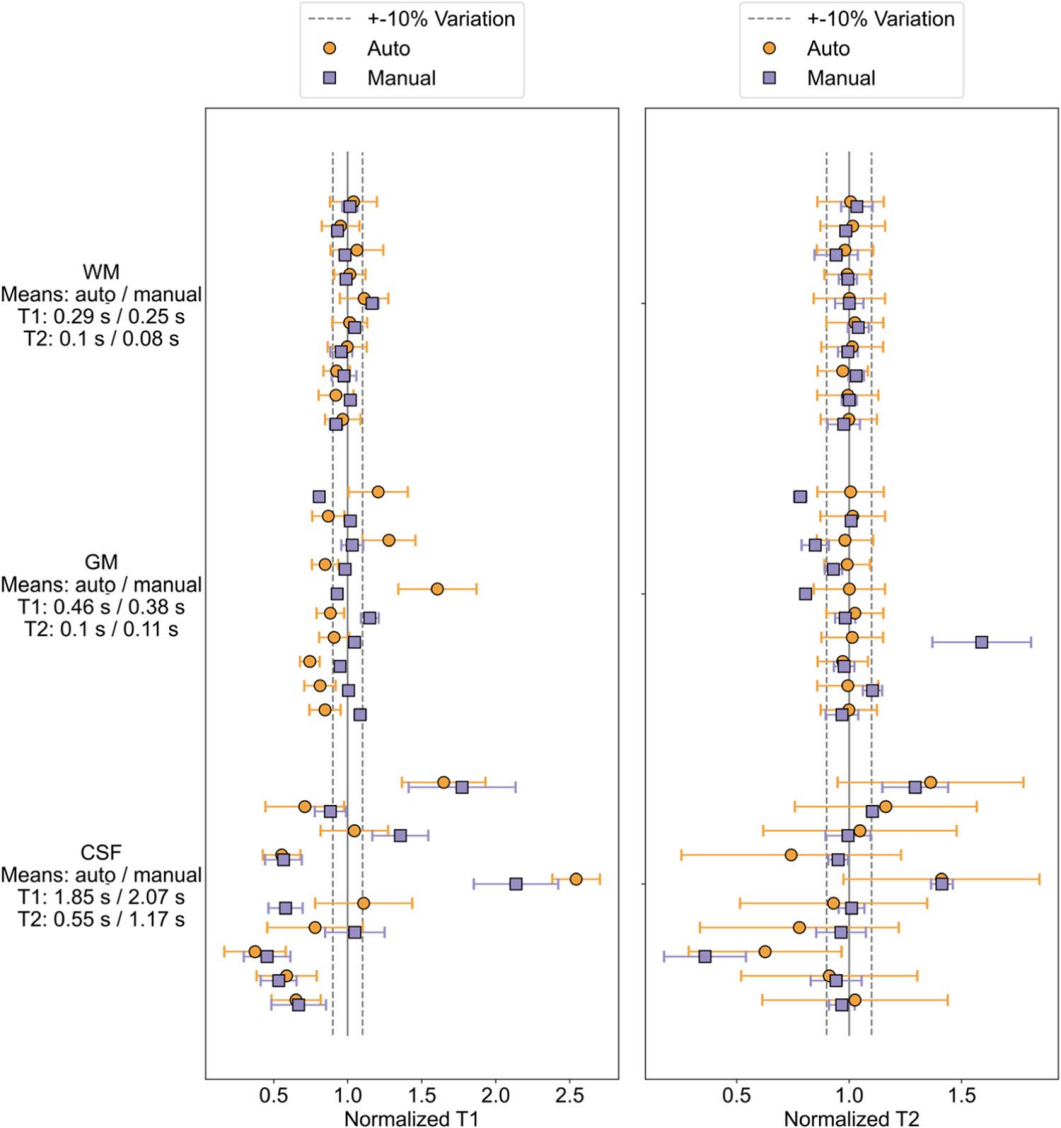
Each volunteer’s ${T}_{1}$ (left) and ${T}_{2}$ (right) mean measurements, with standard deviation shown as error bars. Mean ${T}_{1}$ and ${T}_{2}$ values are shown below tissue labels for auto/manual segmentations

${T}_{1}$ and ${T}_{2}$ measurements normalized to the average measured value for each volunteer and each segmentation type are plotted in Fig. 3. A summary of ${T}_{1}$ and ${T}_{2}$ measurement results for each volunteer and each segmented ROI is shown in the Supplementary Information Table S.2. Averages over all participants for each segmentation type and tissue are given. For ${T}_{1}$, the manual ROI averages are 0.254 s ± 0.0179 s for WM, 0.377 s ± 0.0351 s for GM, 2.073 s ± 1.2007 s for CSF. For ${T}_{2}$, the manual ROI averages are 0.081 s ± 0.0024 s for WM, 0.105 s ± 0.0241 s for GM, 1.172 s ± 0.3234 s for CSF. For ${T}_{1}$, the automatically segmented averages are 0.294 s ± 0.0179 s for WM, 0.46 s ± 0.1258 s for GM, 1.854 s ± 1.2097 s for CSF. For ${T}_{2}$, the automatically segmented averages are 0.097 s ± 0.0016 s for combined WM and GM, 0.553 s ± 0.1422 s for CSF. Two volunteers had manually segmented ${T}_{1}$ ROIs with lower CSF measurements than the automatic segmentations. In one volunteer, the highest CSF ${T}_{1}$ measurement was near the frontal lobe rather than in the ventricles where the manual ROIs were located. In the other, the measured ${T}_{1}$ in the ventricles was the highest of any volunteer, and selecting a square region for the manual ROI could not capture all of the highest ${T}_{1}$ values.

**Fig. 4:**
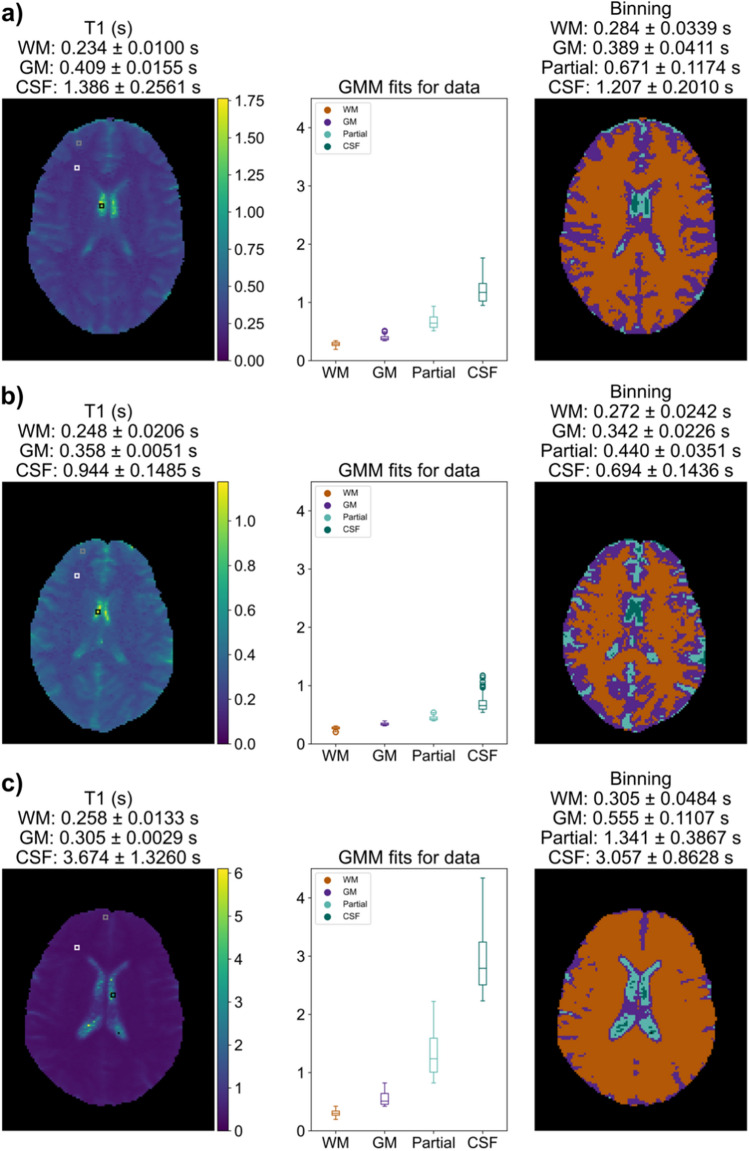
Each row shows one of 3 example ${T}_{1}$ in vivo measurements: (left) ${T}_{1}$ maps with visual indications of manually selected ROIs for WM (white), GM (gray), and CSF (black); (middle) ${T}_{1}$ values by auto-segmented bin; (right) visual map of the auto-segmented bins. Mean and standard deviation of ${T}_{1}$ are indicated for each manual or auto-segmented ROI. **a** A subject exhibiting a typical ${T}_{1}$ map for this study. **b** A subject with small head size showing lower CSF values, likely due to partial volume effects from smaller ventricles. **c** An example subject with large ventricles and a higher CSF measurement

**Fig. 5:**
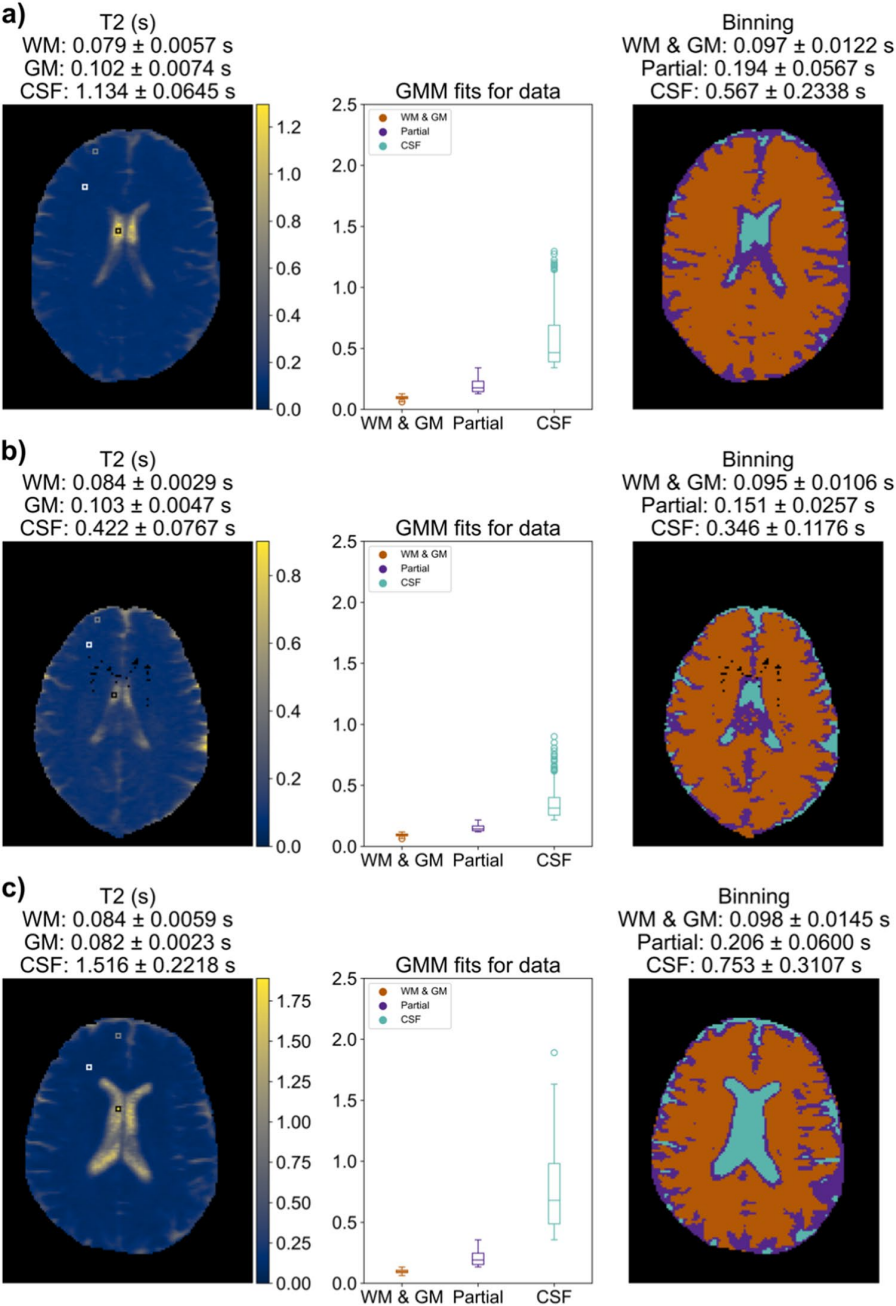
Each row shows one of 3 example ${T}_{2}$ in vivo measurements for the same volunteers of Fig. 4: (left) ${T}_{2}$ maps with visual indications of manually selected ROIS for WM (white), GM (gray), and CSF (black); (middle) ${T}_{2}$ values by auto-segmented bin; (right) visual map of the auto-segmented bins. Mean and standard deviation of ${T}_{2}$ are indicated for each manual or auto-segmented ROI. **a** A subject exhibiting a typical ${T}_{2}$ map for this study. **b** A subject with small head size showing lower CSF values, likely due to partial volume effects from smaller ventricles. **c** An example subject with large ventricles and a higher CSF measurement

Variations were observed across volunteers in head size, ventricle size, the range of measured ${T}_{1}$ and ${T}_{2}$, and automatic segmentation regions (Figs. 4 and 5). A volunteer with typical outcomes is shown (Figs. 4a and 5a). A volunteer with the smallest head size by voxel count (subject 3 of Supplementary Information Table S.2) has lower CSF measurements compared to other volunteers (Figs. 4b and 5b). A volunteer with qualitatively larger ventricles shows higher CSF measurements compared to other volunteers (Figs. 4c and 5c).

**Fig. 6:**
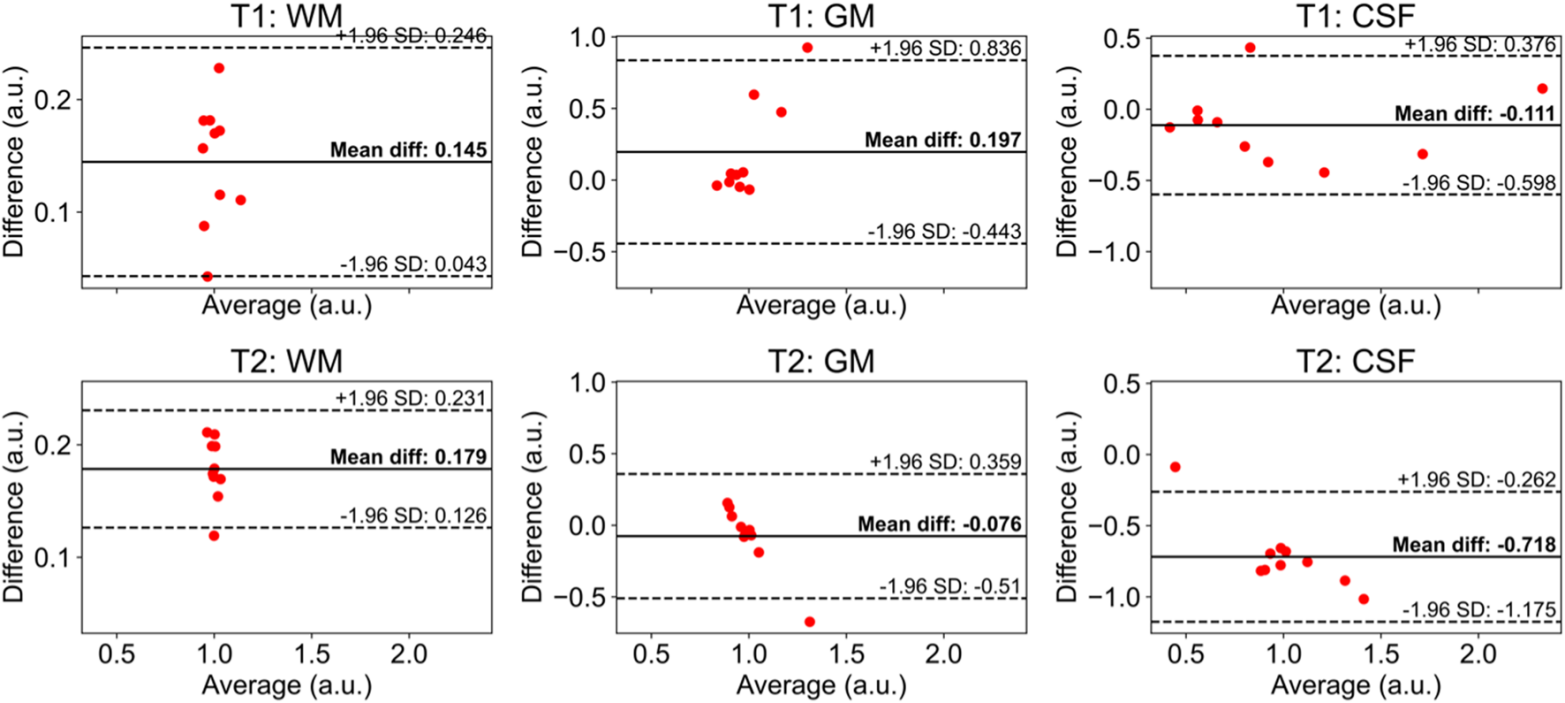
Bland–Altman plots for in vivo measurements using the manual ROIs compared to the automatic segmentations. The differences are calculated as the automatic segmentation measurement minus the manual ROI measurement. Mean differences are marked as the black line and ± 1.96 standard deviations from the mean difference are indicated with dashed lines

Differences in in vivo tissue ${T}_{1}$ and ${T}_{2}$ measurements between manually selected ROIs and automatic segmentations were explored using Bland–Altman plots (Fig. 6). ${T}_{2}$ for GM has mean differences of less than 10% between the two ROI creation methods. Other tissues all have less than 20% mean difference between the methods, except for CSF ${T}_{2}$, which has 72% mean difference between the measurements. Finally, the variance in the differences between the ROI creation methods are smaller for WM and GM than for CSF.

## Discussion

In vivo relaxation properties at 0.064 T were measured for 10 adult volunteers. The protocols used to measure relaxation were optimized when possible, and their accuracy was examined using test samples measured on a 0.064 T NMR system.

### Measurement protocols

${T}_{1}$ And ${T}_{2}$ measurement protocols suitable for in vivo use were developed and validated. The ${T}_{1}$ in vivo protocol was designed to minimize expected error in measured ${T}_{1}$, while keeping the total scan time to be one hour. Simulations of protocols that used fewer $TI$ s resulted in overall worse ${T}_{1}$ estimation than when using additional $TI$ s. However, the scan time limitation required the use of a shorter $TR$ when the number of $TI$ s was increased. Overall, this resulted in a ${T}_{1}$ protocol that can have an ill-conditioned fit for high values of ${T}_{1}$ when the noise in the image is large. Thus, a tradeoff was made between scan time and expected accuracy in high ${T}_{1}$ measurements, resulting in a higher coefficient of variation for the CSF ${T}_{1}$ measurements compared to WM and GM. A limitation to this study is that the ${T}_{1}$ fitting protocol did not account for Rician noise in the IR magnitude images, which can improve fitting outcomes [22, 23].

Test samples were used to validate both of the ${T}_{1}$ and ${T}_{2}$ in vivo MRI protocols compared to an NMR measurement, as well as a reference MRI protocol for ${T}_{1}$. Test samples demonstrated accuracy in the WM and GM range of values but underestimated large ${T}_{2}$ in the CSF range. A limitation of this study is the underestimation of ${T}_{2}$ using the MRI in vivo protocol for CSF, and care should be taken when interpreting CSF ${T}_{2}$ results. There are a few confounds that could influence the accuracy of the long ${T}_{2}$ measurements. Spurious signal pathways due to system imperfections are more likely to compound in long ${T}_{2}$ species, making them more susceptible to errors. Furthermore, the fast spin echo acquisition that was modified to build the ${T}_{2}$ mapping sequence was designed to generate clean clinically useful images in harsh electromagnetic environments. It is not clear how the proprietary steps necessary to ensure good clinical image quality would affect the expected signal decay. Finally, magnitude images were used for the ${T}_{2}$ fit which could bring a Rician noise influence into the data. The ${T}_{2}$ model could be modified to account for Rician noise, or to include a constant term in the fitting. These modifications were not explored in this study.

### In vivo measurement results

Measured in vivo relaxation values can be compared to relaxation values reported in literature for 0.05 T [18], namely for ${T}_{1}$: 0.275 s for WM, 0.327 s for GM, 3.695 s for CSF; and for ${T}_{2}$: 0.102 s for WM, 0.102 s for GM, 1.584 s for CSF. The automatic segmentation results for WM and GM of this study are closer to previous literature results than the manual ROIs, with ${T}_{1}$ of 0.294 s for WM and 0.46 s for GM, and ${T}_{2}$ of 0.097 s for WM and GM.

As mentioned previously, the ${T}_{2}$ measurements for CSF are likely underestimations of the true values due to limitations of the measurement protocol. However, partial volume effects from the 5 mm slice thickness may also cause underestimations in both the ${T}_{1}$ and ${T}_{2}$ measurements. Challenges in accurately measuring CSF are known: earlier studies underestimated ${T}_{1}$ relaxation in CSF due to repetition time settings that were too short [16, 17]. To remove partial volume effects, more recent work at 0.05 T developed a separate protocol specifically targeted to isolate signal when measuring relaxation in CSF [18]. Future work is needed to accurately measure relaxation in CSF at 0.064 T.

### In vivo skull stripping and tissue selection methods

A skull stripping protocol was developed and demonstrated to work effectively at removing background noise and the skull from in vivo quantitative maps. To calculate average ${T}_{1}$ and ${T}_{2}$ times for different tissues, two tissue selection methods were compared. One was an automatic segmentation method and the other used manually selected ROIs. Similar to previous work at a similar field strength [18], we found that the relatively small difference between WM and GM relaxation parameters made it difficult to segment and analyze the two tissues independently, especially using our automatic segmentation method. An improvement to the study would be to acquire anatomical images during scanning; however, for this study it was not possible to acquire anatomical images during the ${T}_{1}$ protocol due to scan time limitations.

To compare average relaxation times using the two tissue selection methods, Bland–Altman plots were used. A large mean difference was observed between the tissue selection methods for CSF ${T}_{2}$, likely because the highest region of measured ${T}_{2}$ was targeted when creating manual ROIs. While the manually selected ROIs could introduce selection bias, the automatic segmentation method could also be biased because the binning method uses the underlying ${T}_{1}$ and ${T}_{2}$ measurements, thus, tissue designation is determined from the relaxation parameter itself in the automatic segmentation algorithm. Ideally a segmentation method would designate tissue independently from the relaxation measurement of a voxel.

## Conclusion

Tissue relaxation measurements for ${T}_{1}$ and ${T}_{2}$ at 0.064 T have been measured and presented for 10 healthy volunteers. Two ROI selection methods were used for determining mean ${T}_{1}$ and ${T}_{2}$ for the volunteers. Test samples were measured on a 0.064 T NMR and a 0.064 T MRI system, and the measurements were compared. This work will contribute to the growing body of research targeted at measuring and disseminating qMRI properties of the human body for a wide range of field strengths.

## Supplementary Information

Below is the link to the electronic supplementary material.

Supplementary file1 (DOCX 775 KB)

## Data Availability

The authors confirm that the data supporting the findings of this study are available within the article and its supplementary materials.
