# Supplementary material for: In vivo quantitative MRI: T1 and T2 measurements of the human brain at 0.064 T
Source: MAGMA. 2023 May 20;36(3):487–98. doi: 10.1007/s10334-023-01095-x (PMC10386946; doi:10.1007/s10334-023-01095-x)
Supplement: Supplementary file 1 — Supplementary file1 (DOCX 775 KB) [file 10334_2023_1095_MOESM1_ESM.docx]

| **Sample Name** | **T1 (s)** | | | **T2 (s)** | |
| --- | --- | --- | --- | --- | --- |
|  | **NMR** | **MRI - *In vivo* protocol** | **MRI - Reference protocol** | **NMR** | **MRI - *In vivo* protocol** |
| 0.025 mmol/L GdGl_3_-EDTA | 1.246 (0.0146) | 1.384 (0.0325) | 1.342 (0.0079) | 1.272 (0.0326) | 0.901 (0.0211) |
| 0.1% agarose mass concentration (%) | 2.241 (0.0096) | 2.392 (0.0673) | 2.229 (0.1041) | 0.604 (0.004) | 0.597 (0.0154) |
| 0.1 mmol/L CuSO_4_ | 1.695 (0.0188) | 1.743 (0.0493) | 1.731 (0.0862) | 1.901 (0.3131) | 1.007 (0.0346) |
| 0.1 mmol/L GdGl_3_-EDTA in 1.5% agarose mass concentration (%) | 0.436 (0.0042) | 0.438 (0.0071) | 0.438 (0.0038) | 0.068 (0.0007) | 0.056 (0.001) |
| 0.5% agarose mass concentration (%) | 1.846 (0.0101) | 2.027 (0.1016) | 1.947 (0.0798) | 0.244 (0.0012) | 0.183 (0.0015) |
| 0.75 mmol/L CuSO_4_ in 0.25% agarose mass concentration (%) | 0.476 (0.0029) | 0.517 (0.0076) | 0.482 (0.0152) | 0.245 (0.0017) | 0.207 (0.0018) |
| 1 mmol/L CuSO_4_ in 1% agarose mass concentration (%) | 0.315 (0.0044) | 0.341 (0.0059) | 0.326 (0.0041) | 0.094 (0.0044) | 0.088 (0.0025) |
| 2 mmol/L CuSO_4_ | 0.235 (0.0035) | 0.222 (0.0027) | 0.219 (0.0024) | 0.222 (0.0026) | 0.216 (0.0045) |
| 4 mmol/L NiCl_2_ in 1.2% agarose mass concentration (%) | 0.328 (0.0074) | 0.335 (0.0034) | 0.329 (0.0033) | 0.087 (0.0005) | 0.078 (0.0028) |
| Deionized water | 2.475 (0.0223) | 2.555 (0.1022) | 2.351 (0.1474) | 2.193 (0.0829) | 1.21 (0.0417) |

**Table S.1** $T_{1}$ and $T_{2}$ mean and standard deviations for test samples measured for the NMR system, for the MRI system and the *in vivo* protocols. Also included for $T_{1}$ are measurements for the MRI system reference protocol.


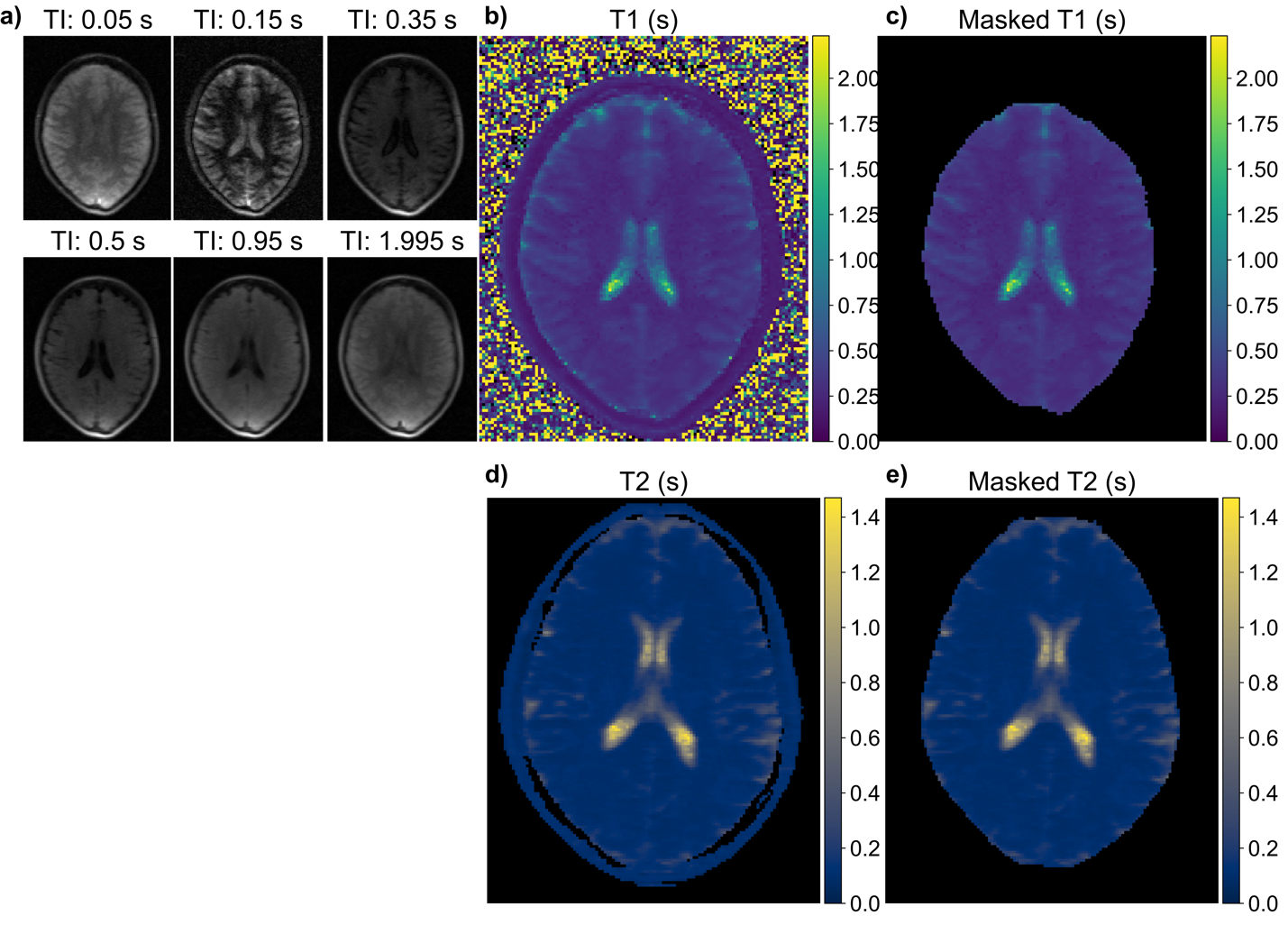


**Fig. S.1** a) Example IR raw data for one subject. b) Example $T_{1}$ map for the subject in (a), and c) shows the result of the skull stripping protocol. d) Example $T_{2}$ map for a different volunteer, with e) the result of the skull stripping protocol.

| **Subject** | **T1 (s)** | | | | | | **T2 (s)** | | | | | **Voxels** |
| --- | --- | --- | --- | --- | --- | --- | --- | --- | --- | --- | --- | --- |
|  | **WM** | | **GM** | | **CSF** | | **WM** | **WM & GM** | **GM** | **CSF** | |  |
|  | **Manual** | **Auto** | **Manual** | **Auto** | **Manual** | **Auto** | **Manual** | **Auto** | **Manual** | **Manual** | **Auto** |  |
| 1 | 0.234 (0.01) | 0.284 (0.0339) | 0.409 (0.0155) | 0.389 (0.0411) | 1.386 (0.2561) | 1.207 (0.201) | 0.079 (0.0057) | 0.097 (0.0122) | 0.102 (0.0074) | 1.134 (0.0645) | 0.567 (0.2338) | 8782 |
| 2 | 0.259 (0.0071) | 0.27 (0.0317) | 0.38 (0.0049) | 0.374 (0.0394) | 1.107 (0.1338) | 1.089 (0.2205) | 0.082 (0.0027) | 0.097 (0.0132) | 0.116 (0.0049) | 1.104 (0.1251) | 0.504 (0.1973) | 8982 |
| 3 | 0.248 (0.0206) | 0.272 (0.0242) | 0.358 (0.0051) | 0.342 (0.0226) | 0.944 (0.1485) | 0.694 (0.1436) | 0.084 (0.0029) | 0.095 (0.0106) | 0.103 (0.0047) | 0.422 (0.0767) | 0.346 (0.1176) | 6917 |
| 4 | 0.243 (0.0179) | 0.293 (0.0387) | 0.395 (0.01) | 0.418 (0.0425) | 2.173 (0.4374) | 1.445 (0.4664) | 0.081 (0.0035) | 0.099 (0.0136) | 0.167 (0.0367) | 1.13 (0.1247) | 0.43 (0.1902) | 7453 |
| 5 | 0.266 (0.0073) | 0.298 (0.0351) | 0.434 (0.0262) | 0.406 (0.038) | 1.203 (0.139) | 2.054 (0.6738) | 0.085 (0.004) | 0.1 (0.0126) | 0.103 (0.0046) | 1.185 (0.0678) | 0.514 (0.2139) | 7631 |
| 6 | 0.296 (0.0132) | 0.326 (0.0534) | 0.351 (0.0127) | 0.738 (0.1946) | 4.43 (1.2622) | 4.716 (0.7551) | 0.082 (0.0051) | 0.097 (0.0154) | 0.085 (0.0011) | 1.655 (0.0804) | 0.779 (0.3398) | 7597 |
| 7 | 0.251 (0.008) | 0.298 (0.0316) | 0.371 (0.0074) | 0.39 (0.0349) | 1.174 (0.1456) | 1.027 (0.13) | 0.081 (0.0032) | 0.097 (0.0098) | 0.098 (0.0038) | 1.115 (0.0484) | 0.41 (0.2004) | 8387 |
| 8 | 0.25 (0.0077) | 0.312 (0.0557) | 0.389 (0.0287) | 0.588 (0.1052) | 2.81 (0.5314) | 1.938 (0.4432) | 0.077 (0.0074) | 0.096 (0.012) | 0.089 (0.0054) | 1.166 (0.1164) | 0.579 (0.2491) | 8676 |
| 9 | 0.237 (0.0086) | 0.28 (0.0356) | 0.384 (0.0121) | 0.399 (0.0435) | 1.831 (0.1938) | 1.317 (0.3497) | 0.08 (0.0019) | 0.099 (0.0142) | 0.106 (0.0023) | 1.294 (0.0317) | 0.643 (0.2597) | 8880 |
| 10 | 0.258 (0.0133) | 0.305 (0.0484) | 0.305 (0.0029) | 0.555 (0.1107) | 3.674 (1.326) | 3.057 (0.8628) | 0.084 (0.0059) | 0.098 (0.0145) | 0.082 (0.0023) | 1.516 (0.2218) | 0.753 (0.3107) | 8309 |
| All | 0.254 (0.0177) | 0.294 (0.0179) | 0.377 (0.0351) | 0.46 (0.1258) | 2.073 (1.2007) | 1.854 (1.2097) | 0.081 (0.0024) | 0.097 (0.0016) | 0.105 (0.0241) | 1.172 (0.3234) | 0.553 (0.1422) | 81614 |

**Table S.2** Per-subject manual and automatic segmentation $T_{1}$ and $T_{2}$ results, with standard deviations shown in parentheses. The number of voxels per slice that were considered within the skull are given. Averages over all subjects are given, along with the total number of voxels measured. Note that for the $T_{2}$ auto-segmentation results, WM and GM were indistinguishable.
